# Landing Proteins on Graphene Trampoline Preserves Their Gas-Phase Folding on the Surface

**DOI:** 10.1021/acscentsci.2c00815

**Published:** 2022-12-14

**Authors:** Kelvin Anggara, Hannah Ochner, Sven Szilagyi, Luigi Malavolti, Stephan Rauschenbach, Klaus Kern

**Affiliations:** † Max-Planck Institute for Solid-State Research, Heisenbergstrasse 1, Stuttgart DE-70569, Germany; ‡ Chemistry Research Laboratory, Department of Chemistry, University of Oxford, 12 Mansfield Road, Oxford OX1 3TA, United Kingdom; § Institut de Physique, École Polytechnique Fédérale de Lausanne, Lausanne CH-1015, Switzerland

## Abstract

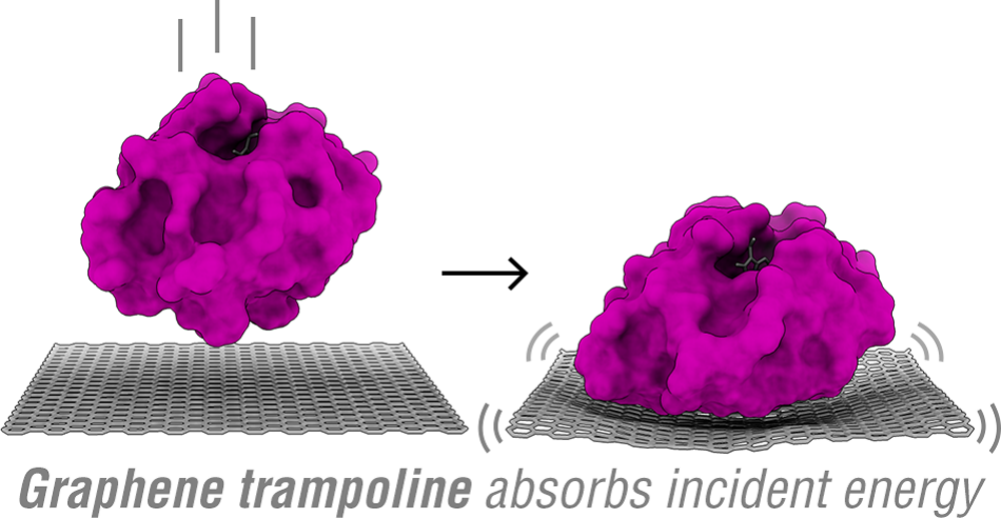

Molecule–surface collisions are known to initiate dynamics that lead to products inaccessible by thermal chemistry. These collision dynamics, however, have mostly been examined on bulk surfaces, leaving vast opportunities unexplored for molecular collisions on nanostructures, especially on those that exhibit mechanical properties radically different from those of their bulk counterparts. Probing energy-dependent dynamics on nanostructures, particularly for large molecules, has been challenging due to their fast time scales and high structural complexity. Here, by examining the dynamics of a protein impinging on a freestanding, single-atom-thick membrane, we discover *molecule-on-trampoline* dynamics that disperse the collision impact away from the incident protein within a few picoseconds. As a result, our experiments and *ab initio* calculations show that cytochrome c retains its gas-phase folded structure when it collides onto freestanding single-layer graphene at low energies (∼20 meV/atom). The *molecule-on-trampoline* dynamics, expected to be operative on many freestanding atomic membranes, enable reliable means to transfer gas-phase macromolecular structures onto freestanding surfaces for their single-molecule imaging, complementing many bioanalytical techniques.

## Abstract 2

Efficient molecule-to-surface energy transfer in the landing of proteins on freestanding graphene preserves their gas-phase folding state on the surface, enabling them to be imaged one at a time.

## Introduction

Molecular collision is one of the simplest ways for a molecule to obtain enough energy to initiate chemical reactions or conformational switches. Examining how energy flows in a molecule during its collision event is critical to understand—and eventually control—the outcome of any molecular collisions.

Molecule–surface collisions are especially important due to the ubiquity of molecule–surface interactions in many areas of chemistry such as heterogeneous catalysis,^1−7^ bottom-up material fabrication,^8−10^ mechanochemistry,^1,11,12^ astrochemistry,^8^ and macromolecular structure characterization.^13−17^ Intricate details of molecule–surface collisions have been revealed by molecule–surface scattering experiments *in vacuo*(1−7) that, for macromolecules, have led to rich applications enabled by the soft and reactive landing of molecules on surfaces.^13^ These studies highlight a unique feature of molecule–surface collision which promptly (sub-picoseconds) converts molecular translational energy toward the surface (*T*) to molecular vibrational energy associated with soft, flexible modes of the molecule (*V*_mol_). Such a prompt energy transfer has been shown to lead to collision outcomes that are thermally inaccessible, such as conformational changes^17,18^ (small *V*_mol_ transferred), mechanochemical reactions,^11^ and molecular fragmentation^14−16^ (large *V*_mol_ transferred), thereby offering a means to manipulate molecular structures.

An important frontier in molecule–surface collision dynamics is the quest to minimize *T* → *V*_mol_ energy transfer in molecule–surface collision, which would preserve the nuclear and electronic state of gas-phase molecules when they are deposited on a surface *in vacuo*. Success in such undertaking will enable gas-phase molecular species to be preserved and immobilized on surfaces and structurally characterized one molecule at a time by single-molecule microscopy methods, such as electron microscopy^19−21^ or scanning probe microscopy techniques.^22^ These new capabilities will unlock new frontiers in both single-molecule microscopy and native mass spectroscopy, as it enables single-molecule visualization of any molecular species relevant to chemistry and biology that can be isolated from the solution phase by native electrospray. Such a combination has been shown for macromolecules by successful soft deposition and direct observation of folded proteins on a surface, ranging from small proteins (∼12 kDa) to large multiunit protein complexes (∼800 kDa).^20,21,23−25^

Realizing the full potential of this vision calls for strategies to divert the flow of molecular translational energy (*T*) away from the molecular internal coordinates in molecule–surface collision. One ideal destination for the energy is the surface, since it possesses a large number of soft vibrational/phonon modes (*V*_surf_), well suited to absorb the translational energy of the incident molecule. Collision dynamics dominated by *T* → *V*_surf_ energy transfer would minimize the energy transferred to the internal coordinates of the molecule and preserve the gas-phase molecular structure on surface that can be subsequently characterized by many surface-based microscopy techniques.^19−22^ Given that the translational energy (*T*) would flow to the *softest mode* between the collision partners,^26,27^ be that mode residing in the molecule or the surface, replacing *T* → *V*_mol_ by *T* → *V*_surf_ requires the surface to possess softer vibrational modes than the incident molecule. This requirement has been fulfilled in previous studies by noble-gas^28,29^ or organic adlayers^30−32^ on bulk surfaces, which cushion the impact of incident molecules on surfaces.^28−34^

Here we point out that freestanding atomic membranes, such as single-layer graphene (SLG), possess a soft “trampoline” mode that fulfills the requirement for *T* → *V*_surf_ dynamics. We demonstrate experimentally the use of freestanding graphene as the landing surface for cytochrome c (cyt c) proteins, which preserves the gas-phase folding state of cyt c on the surface. Ions of cyt c were generated by nanoelectrospray^35,36^ (nESI) and soft-landed on graphene *in vacuo* by an electrospray ion beam deposition (ESIBD) technique,^13,37^ whereupon the adsorbed cyt c structure was imaged one molecule at a time by low-energy electron holography^20,21^ (LEEH). We corroborate our experimental findings by simulating the collision dynamics of the cyt c on graphene using *ab initio* molecular dynamics (AIMD) implemented in density functional theory (DFT). Our simulation shows the dominance of *T* → *V*_surf_ dynamics in the landing of molecules on the freestanding atomic membrane, where *V*_surf_ is the soft acoustic vibrations of graphene. The efficient protein to graphene energy transfer preserves the gas-phase folding state of the protein on the surface, as observed in the experiment. The *T* → *V*_surf_ dynamics on a freestanding atomic membrane, here termed *molecule-on-trampoline* dynamics, are expected to be operative in the encounter of rigid molecules with many freestanding 2D materials,^38^ such as graphene oxide, hexagonal boron nitride, metal chalcogenides (e.g. MoS_2_), etc. We focus our present work on graphene due to its widespread use across scientific disciplines that span from fundamental physics^39^ to structural biology.^40^ Our work shows the potential of the tandem combination of nESI + ESIBD + LEEH on graphene to reveal, at the single-molecule level, folded structures of molecular species generated from the native mass spectroscopy techniques.

## Results and Discussion

**Figure 1:**
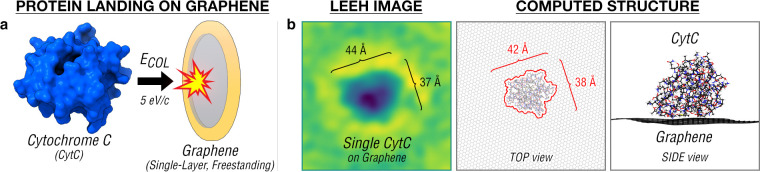
Landing folded proteins on freestanding single-layer graphene. (a) Cytochrome c (cyt c) [M+7H]^7+^ ions are landed normal to a freestanding single-layer graphene with a selected collision energy of 35 eV (i.e. 5 eV/charge, typical for soft-landing of molecules on a surface). (b) Folded cyt c on graphene imaged by low-energy electron holography (LEEH) showing agreement with folded cyt c computed from density functional theory (DFT). The shape of cyt c from the DFT calculations is given by the red outline obtained from the van der Waals representation of the protein. The deposition and imaging were done at room temperature.

Figure 1a shows a schematic of the experiment where cyt c ions ([M+7H]^7+^) were collided normal to a freestanding graphene held at room temperature. The proteins were collided at a selected energy of 35 eV collision energy (i.e. 5 eV/charge), previously shown to preserve the chemical structures of incident molecules on surfaces.^13,41^ The proteins adsorbed on graphene were subsequently imaged as single molecules using LEEH to reveal their structures, as inferred from their observed sizes and shapes.

The cyt c protein soft-landed on graphene was observed by LEEH as a folded protein (Figure 1b). The observed dimension of cyt c on graphene was found to agree well with the dimension of folded cyt c obtained by relaxing the final state of our AIMD calculations (Figure 1b). In addition, the measured size of cyt c on graphene with an apparent area of ∼13 nm^2^ was found to agree with the size of folded cyt c measured in the gas phase by ion-mobility experiments^42^ (∼14 nm^2^). Given the low energy involved in the landing event,^13^ the folding state of the adsorbed cyt c is expected to be similar to its gas-phase folded structure, consistent with the previous protein landing studies on graphene.^20,21^

**Figure 2:**
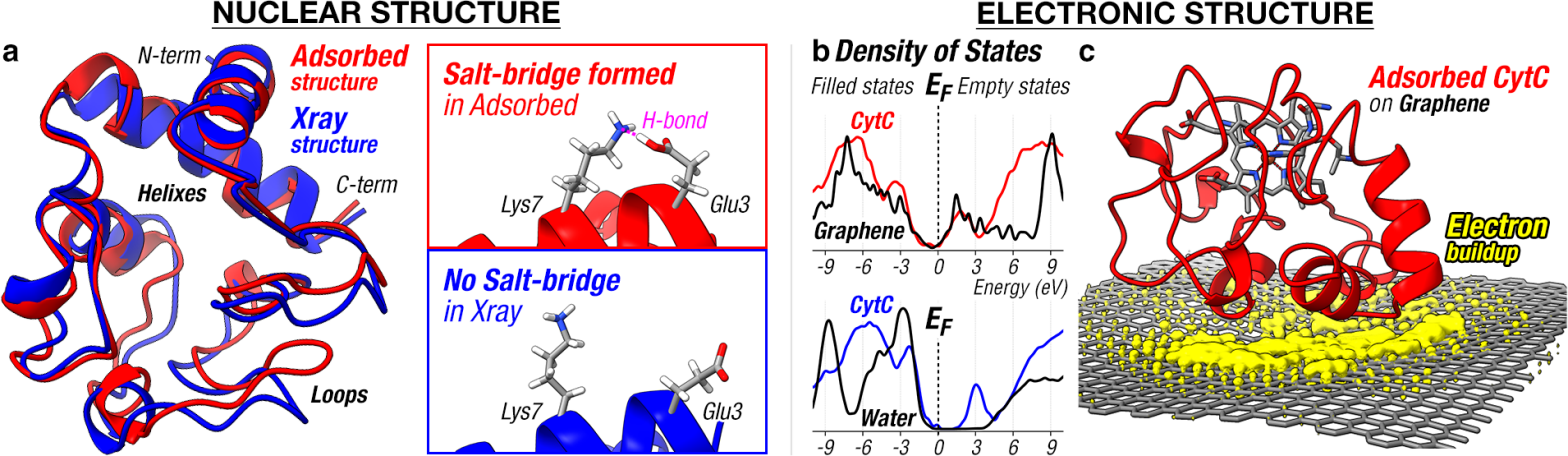
Nuclear and electronic structure of a protein on single-layer graphene. (a) cyt c adsorbed on graphene (red) is computed to be structurally similar to its crystal structure (blue, PDB ID 1HRC). The adsorbed cyt c structure when compared to its crystal structure possesses more “salt bridges” between the positively and negatively charged amino acid side chains. (b) Projected density of states (pDOS) calculations show strong overlap between cyt c and graphene electronic states, indicating an efficient path of molecule-to-surface electron/hole transfer. Comparatively, for hydrated cyt c, some unoccupied cyt c states do not overlap with water electronic states, indicating that electrons could be trapped in these states. (c) The positively charged cyt c attracts the graphene electrons into a pool underneath the protein (yellow density, isosurface 2 × 10^–3^ e Å^–3^) to establish charge–image charge interactions.

We corroborate our findings by investigating the structural changes in the protein when it lands from the gas-phase onto the surface, using AIMD calculations at the level of DFT. We model the landing event by colliding a gas-phase cyt c^7+^ protein ion onto a freestanding graphene, whereby the protein possesses a 35 eV translational energy toward the graphene. We approximate the gas-phase structure of cyt c^7+^ ion by relaxing the crystal structure of cyt c (PDB ID 1HRC) in the gas phase with a net charge of +7. Our calculation gives a gas-phase cyt c structure that has a near-identical three-dimensional structure to its crystal structure, with the most significant changes being localized at the protein–vacuum interface. These structural changes are caused by the formation of salt bridges and hydrogen bonds among the side chains of the amino acids at the protein–vacuum interface, thereby confirming the “side chain collapse” in the literature^43−46^ at the *ab initio* level (Figure 2a, inset). These cohesive interactions at the protein–vacuum interface are understood to cause a surface-tension-like effect^47^ that maintains the folded structure of the protein in the gas phase. The gas-phase cyt c^7+^ structure from our calculations yields an ion-mobility cross section (∼12 nm^2^, from the IMPACT software^48^) that agrees well with its measured experimental value^42^ (∼14 nm^2^). The slight difference between theory and experimental cross sections may be attributed to the finite temperature effects, such as cyt c structural dynamics at ∼300 K, that have yet to be included in present DFT calculations.

Our AIMD calculations, in agreement with the experiment, show that the principal features of the gas-phase cyt c are preserved when the protein lands on the surface. The AIMD calculation gives a final structure of cyt c on graphene whose size and shape agree well with the adsorbed cyt c observed by LEEH (Figure 1b). The computed structure of adsorbed cyt c has a near identical folding state to its crystal structure (PDB ID 1HRC) with the most significant changes located at the omega loops of the protein (residue 36 to 37, 44 to 46, 54 to 60, and 76 to 77) (Figure 2a). Our finding echoes the cyt c foldon hierarchy^49^ that regards the omega loops in cyt c as the most flexible part of the molecule and thus the structural motif that responds most to changes in the external environment of the protein.

Our *ab initio* calculations on the entire protein shed light into the electronic structure of cyt c on graphene, revealing its density of states and its binding mechanism to the surface. Figure 2b shows the projected density of states (pDOS) of the adsorbed cyt c and the underlying graphene, revealing a strong overlap between the cyt c and graphene electronic states. Such a strong overlap provides an explanation for the remarkable stability of the proteins on graphene from the continuous ∼100 eV electron irradiation in the LEEH imaging (i.e. no event with the probability higher than ∼10^–12^ per incident electron was observed; see Methods for details). Given that low-energy electrons (50–150 eV) are known to efficiently ionize molecules (M + e → M^+^ + 2e) and cause molecular dissociations,^50−52^ the apparent immunity of proteins on graphene toward electron-induced dissociations indicates an efficient hole removal from the protein. This efficient hole transfer mechanism is understood to originate from the large electronic transition probability between the protein and graphene, enabled by the strong electronic state overlap between them—a common feature for molecules adsorbed on conductive surfaces.^53^ For molecules adsorbed on metal surfaces,^54,55^ the lifetime of transient charges trapped in the molecules was measured to be below 5 fs—a time scale that is too short for any molecular bonds to significantly stretch to cause any chemical reactions, which typically requires tens or hundreds of femtoseconds.^56^ This insight highlights the importance of fast electronic relaxation in suppressing electron-induced reactive events in adsorbed molecules.

We contrast the cyt c-graphene pDOS to that for hydrated cyt c (Figure S1), which shows the nonoverlap between unoccupied cyt c states and the water electronic states between the Fermi level to ∼3 eV above the Fermi level (Figure 2b, lower panel). These “unprotected” cyt c states consist of the π-states in the heme, the nitrogen base, and the peptide bonds of the protein (Figure S2). The nonoverlap implies that electrons can be trapped on these “unprotected” states to cause molecular dissociations, since these trapped charges can only be removed by slow nuclear dynamics (e.g. a Grothuss-like mechanism^57^) as opposed to fast electronic dynamics. Our comparison thereby highlights the importance of protein contact to a conductive medium (e.g. graphene) in providing fast electronic de-excitation pathways for the protein.

The binding of proteins to graphene is driven by noncovalent (electrostatic and van der Waals) interactions, as revealed by our calculation that shows that every C atom on the graphene retains its C-sp^2^ geometry. Our calculations estimate the cyt c–graphene binding energy to be ∼33 eV, originating primarily from electrostatic interactions (77%) and secondarily from van der Waals interactions (23%). The dominant electrostatic cyt c–graphene interaction is understood to arise from the net positive cyt c (+3.2e) accumulating a pool of electrons on the underlying graphene (−3.2e) that leads to a charge–image charge interaction between cyt c and graphene (Figure 2c). The positive charge in the adsorbed cyt c originates from all the retained protons that are initially attached to the protein in the gas phase. These protons are likely to remain attached to the protein due to their high detachment barriers, computed to be above +0.9 eV if detached from histidine and above +1.4 eV from lysine. Given that our relaxation calculations show that all protons initially attached to the protein in the gas phase remain attached to the adsorbed protein, we note that the computed +3.2e net charge on the adsorbed protein is lower than the +7e charge in the gas-phase protein. This charge difference thereby indicates a significant graphene-to-protein electron injection during the landing of the protein on the surface.

**Figure 3:**
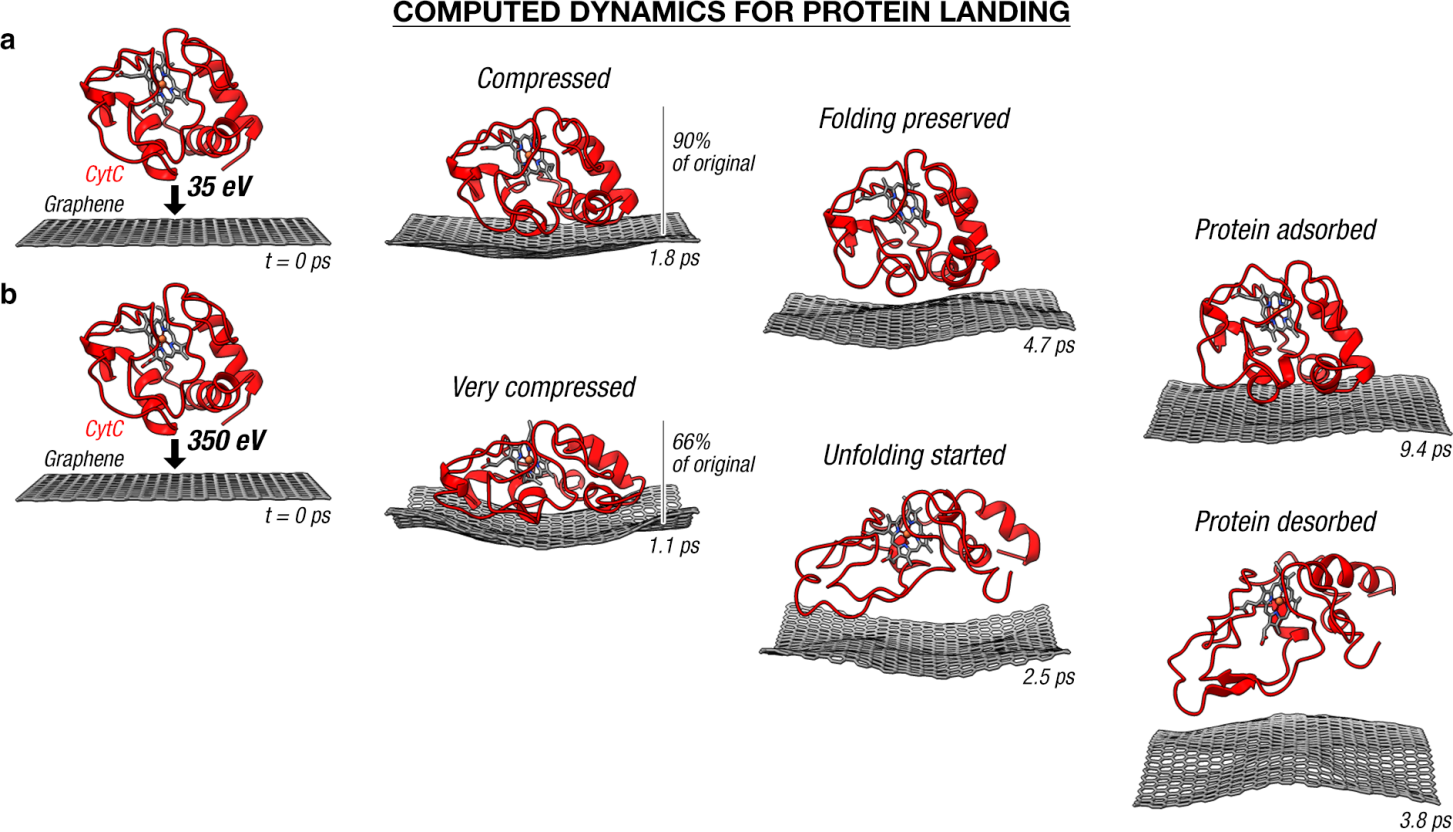
*Ab initio* molecular dynamics of protein landing on single-layer graphene. Folded cyt c is landed on graphene with an energy of 35 eV (i.e. 5 eV/charge—typical for soft-landing), shown in (a), and 350 eV (i.e. 50 eV/charge—typical for reactive-landing), shown in (b). The collision compresses the incident protein, initiating the protein unfolding in the latter case. The graphene has traveled downward by 3.1 nm at the 9.4 ps mark in (a) and by 4.7 nm at the 3.8 ps mark in (b); these distances are the greatest distance traveled by graphene in their respective trajectories.

We now detail the mechanism that connects the initial, gas-phase protein structure to the final, adsorbed protein structure. Figure 3a shows the soft-landing dynamics of cyt c on graphene, as per our experiment, at 35 eV collision energy (i.e. 5 eV/charge) and at an approach angle normal to the surface. The calculation shows that landing a protein at such energy on graphene preserves its primary structure and most of its secondary and tertiary structure from the gas phase to the surface, consistent with the conclusions from the experiment. The computed dynamics show that the protein–surface collision creates a soliton-like collective oscillation in the protein, which propagates as fast as ∼2.5 nm/ps away from the surface (Figure S3a), similar to the previously reported “proteinquake” collective oscillation in a protein.^58,59^ This oscillation subsequently is followed by a slight compression (∼10%) and decompression of the entire protein prior to its stable adsorption on graphene (Figure 3A and Figure S4a).

The protein compression evidences the conversion of its molecular translational energy toward its molecular vibrational energy. Specifically, the most flexible, collective vibrational modes of the protein are excited upon landing, whose magnitude dictates the extent of structural change in the protein upon its landing on surface. We verify this claim by comparing AIMD calculations of cyt c at soft landing (35 eV, 5 eV/charge, Figure 3a and Figures S3a and 4a) and reactive landing regimes (350 eV, 50 eV/charge, Figure 3b and Figures S3b and 4b). In the low-energy regime, the gained vibrational energy is insufficient to cause major conformational changes, as shown by the similar protein folding between the final adsorbed structure and the crystal structure (Figure 2a). In the high-energy regime, the gained vibrational energy is sufficient to alter the tertiary structure of cyt c to initiate the unfolding of the protein while some secondary structures (i.e. the helices) remain intact (Figure 3b). The surface-induced protein compression causes a prompt (within 1–2 ps) and specific structural perturbation on the protein that could lead to thermally inaccessible products/outcomes, such as, in the case for small molecules, bond-selective mechanochemical reactions,^11^ or in the case of macromolecules, conformation changes^27^ and fragmentations.^14−16,31,32^ We thereby argue that the surface-collision-induced compression is the key to access unique fragmentation pathways observed in the surface-induced dissociation (SID) technique^15,16^ that uses a high-energy collision of macromolecules on surfaces to induce their fragmentations for macromolecular structure characterization.

**Figure 4:**
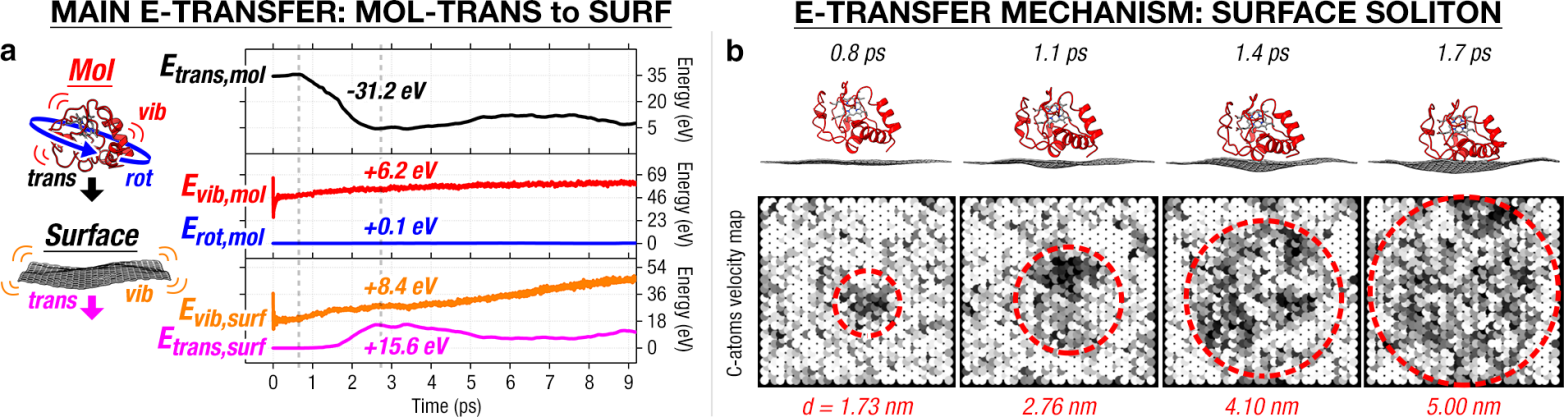
Quantitative analysis of protein landing dynamics on single-layer graphene at 35 eV collision energy. (a) A protein–graphene collision principally converts the protein translational energy (*E*_trans,mol_) to the graphene vibrational energy (*E*_vib,surf_ and *E*_trans,surf_). The graphene translational energy (*E*_trans,surf_) would approximate the energy received by the “trampoline” mode of the graphene. The sum of *E*_vib,surf_ and *E*_trans,surf_ would give the total vibrational energy of graphene (termed *V*_surf_ in the main text). (b) A protein–graphene collision creates a soliton in the graphene that serves as a mechanism to promptly transport energy away from the landing site, as fast as ∼3–4 nm/ps (black atoms denote C atoms moving away from the protein).

An energy analysis of protein landing on graphene reveals the dominance of *T* → *V*_surf_ energy transfer as its most striking feature (Figure 4 and Figure S5). Figure 4a shows that the molecular translational energy (*T*) is converted largely to the surface vibrational energy (*V*_surf_) (77%) and, to a lesser extent, to the molecular vibrational energy (*V*_mol_) (19%). These results are in stark contrast to that for molecular landing on bulk surfaces,^11,18^ whereby *T* is converted primarily to *V*_mol_ (34%), and secondarily to *V*_surf_ (23%). The different energy transfer dynamics on *hard*, bulk surfaces and *soft*, freestanding graphene echo the previously reported experiments^31,32^ and calculations^33^ of peptides scattering on *hard*, fluorinated self-assembled monolayers (SAMs) and *soft*, hydrocarbon SAMs. We additionally note that these dynamics also minimize the *T* → Rmol energy transfer (*R*_mol_ = molecular rotational energy), which suggests that the observed molecular orientation on the surface would be largely similar to the orientation of the gas-phase molecule prior to its landing. Protein rotations only become significant in the collision dynamics at very low collision energy, such as at 3.5 eV (Figure S6), because the slowly moving protein has ample time to experience the attractive forces from the surfaces that reorients the protein throughout its surface collision (also known as “the dynamic steering effect”^60^).

The dominance of *T* → *V*_surf_ in a protein–graphene collision evidences a coupling between incident protein translation and graphene vibration. Specifically, our calculations show the collision exciting the out-of-plane, soft “trampoline” vibrational mode in graphene, whose fundamental is measured to be in megahertz^61,62^ due to the low mass and the freestanding state of graphene. The protein–graphene collision generates a soliton-like, traveling transverse wavepacket in the graphene (Figure 4b) (also called a stress wave^63^ or an elastic deformation wave^64^), understood to be the superposition of various out-of-plane modes of freestanding graphene. At short time scales (approximately picoseconds) covered by our AIMD calculations, our result shows that the soliton travels as fast as 3–4 nm/ps, in agreement with previously computed values.^65^ At long time scales (approximately nanoseconds to approximately milliseconds), we expect the soliton to gradually disperse into various out-of-plane modes of the graphene,^66^ as its kinetic energy equipartitions to all degrees of freedom in the graphene. The soliton dynamics highlight the fast protein-to-surface energy dissipation channel for the protein translational energy, which enables the preservation of protein folded states when they land on freestanding surfaces. These *molecule-on-trampoline* dynamics are expected to be general in the collision of rigid molecules with many freestanding 2D materials, which opens up new research opportunities at the intersection of reaction dynamics, macromolecular chemistry, nanoelectromechanical (NEMS) resonators,^67^ and 2D materials research. One interesting avenue is to examine the scaling behavior of these dynamics across different projectile sizes, starting from a small molecule (e.g. amino acid) to a large macromolecular complex (e.g. membrane proteins in a micelle), and to examine whether their surface encounters could be discerned by transient electrical transport measurements.^68^

## Conclusions

This work provides important physical descriptions of protein nuclear and electronic structures when they are isolated *in vacuo* and adsorbed on graphene, critical to assess new opportunities afforded by the tandem combination of native electrospray ionization, soft-landing technology, and single-molecule microscopy techniques. Our experiments and *ab initio* calculations reveal a fast protein-to-surface energy transfer mechanism in a protein–graphene collision that allows the gas-phase protein structures to be preserved at a freestanding surface. We point out that surface collision on an atomic membrane could provide a means to access the ground and excited conformational states of macromolecules via their compressions. For molecular collisions on organic adlayers,^30^ we anticipate that a similarly effective energy dissipation mechanism involving different surface modes^33,34^ may be operative to preserve the gas-phase protein structures on these surfaces. Observing these gas-phase structures on surface one at a time by single-molecule microscopy techniques would complement the structural studies conducted by ion-mobility and native mass spectroscopy techniques. This single-molecule approach could prove particularly valuable for small, flexible proteins, glycans, or glycoproteins, which are difficult to observe by ensemble-averaged approaches.

## Methods

### Experiment

Cytochrome c (cyt c) (Sigma-Aldrich, >95%, from Equine Heart, Catalog Number C7752) was dissolved in a 200 mM ammonium acetate solution and desalted twice (Bio-Rad BioSpin P6 column) to give a cyt c spray solution (1.5 mg/mL). The solution was loaded to a metal-coated nanoelectrospray glass emitter and sprayed at 1.0–1.5 kV to a 80 °C capillary inlet to prevent denaturation of the protein. No unexpected or unusually high safety hazards were encountered. Using an electrospray ion beam deposition (ESIBD) setup, described in detail elsewhere,^21,37^ the ions were mass-selected to yield an ion beam that consisted of +7 (major, *m*/*z* = 1764) and +8 (minor, *m*/*z* = 1543) cyt c ions and subsequently deposited on a freestanding single-layer graphene (SLG) held at room temperature under ultrahigh vacuum (*P* < 10^–10^ mbar). The protein–graphene collision energy was controlled by applying a selected voltage to decelerate the incident protein ion. After the deposition, the graphene sample was transferred *in vacuo* to a low-energy electron holography (LEEH) instrument, operated at room temperature under ultrahigh vacuum (*P* < 10^–10^ mbar). Low-energy electrons between 50 and 150 eV were used in LEEH imaging with a dose estimated at ∼10 nA per 50 nm^2^. Illuminating a protein for ∼1 s was sufficient to record a high-quality hologram, which was subsequently reconstructed using the previously described method^21^ to reveal the real-space image of the adsorbed protein.

The cyt c protein shown in Figure 1b was observed continuously for 23 s using an electron energy of 147 eV. The protein structure was observed to remain unchanged throughout the illumination time, during which a total of ∼4 × 10^11^ electrons had been scattered on the protein. Given that no inelastic event (e.g. diffusion, rotation, or dissociation) was observed, our data thereby set an upper limit for any inelastic event probability to be ∼3 × 10^–12^ event per electron.

### Theory

*Ab initio* calculations at the level of density functional theory (DFT) as implemented in the code OpenMX^69−71^ (version 3.9.2) was used to model the experiment. The code employed norm-conserving pseudopotentials, pseudoatomic localized basis functions, periodic boundary conditions, and van der Waals correction using Grimme’s DFT-D3 method.^72^ Our calculations employed a cutoff energy of 300 Ry, an electronic temperature of 300 K, and a supercell with dimensions of 51.33 Å (*X* axis), 49.39 Å (*Y* axis), and 50.00 Å (*Z* axis). All calculations sampled only the gamma point of the *k*-mesh and used an electronic convergence criterion of 4 × 10^–8^ hartree, employing the divide-conquer with localized natural orbitals (DC-LNO) method.^73^ The graphene was modeled by 960 C atoms, and the cyt c was modeled with a total of 1744 atoms as C_558_H_878_O_155_N_148_FeS_4_. The relaxation calculations were performed until the forces in all atoms were below 4 × 10^–4^ hartree/bohr. Charge analyses of the relaxed structures were performed using a Bader analysis.^74^ Visualization of the molecular structures was performed using the ChimeraX software.^75,76^

Born–Oppenheimer MD calculations were performed as a microcanonical ensemble that preserved the total number of atoms (*N*), volume (*V*), and energy (*E*). The MD calculations were performed with a 0.5 fs time step, which gave a negligible total energy drift of as low as ∼2 meV/ps per atom. The initial state of the MD placed a gas-phase relaxed cyt c ∼7 Å above the graphene. All atoms were subsequently initialized with random velocities sampled from the Boltzmann distribution at room temperature (298 K). The thermalization of these velocities is evident in the first few femtoseconds at the start of the trajectory, shown in the *E*_vib,mol_ and *E*_vib,surf_ of Figure 4a and Figure S5a. On top of these velocities, all atoms in cyt c receive a constant velocity toward the graphene that corresponded to the translational energy of the protein toward the graphene of either 35 or 350 eV. In the MD calculations, all of the C atoms of the graphene were free to move, causing the act of restoring force in the graphene “trampoline” onto the protein to be unaccounted for in the MD calculations. However, these forces, estimated to be ∼0.3–0.6 nN from ref (61), were minor in comparison to the forces experienced by the protein during the collision with the graphene at ∼10 nN for 5 eV/c landing and ∼50 nN for 50 eV/c landing (Figure S4) and thereby were not expected to be the major factor in the landing dynamics of the protein on graphene.

## Data Availability

All data required to evaluate the conclusions of the paper is present in the main text or the Supporting Information. Supporting Information contains analysis of hydrated cyt c^7+^ and cyt c^7+^ colliding with graphene. The AIMD trajectories for cyt c landing on graphene, as well as the atomic coordinates for the gas-phase cyt c^7+^, hydrated cyt c^7+^, and the adsorbed cyt c on graphene are available at the Data Repository of the Max Planck Society (10.17617/3.VJ9ZIM).

## Supporting Information Available

Structural analysis of hydrated cyt c^7+^ and impulse analysis of cyt c colliding on graphene (PDF)
Transparent Peer Review report available (PDF)
