# Supplementary material for: Landing Proteins on Graphene Trampoline Preserves Their Gas-Phase Folding on the Surface
Source: ACS Cent Sci. 2022 Dec 14;9(2):151–8. doi: 10.1021/acscentsci.2c00815 (PMC9951278; doi:10.1021/acscentsci.2c00815)
Supplement: Supplementary file 1 — oc2c00815_si_001.pdf [file oc2c00815_si_001.pdf]

SUPPORTING INFORMATION

Landing Proteins on Graphene Trampoline Preserves their Gas-Phase Folding on Surface

Kelvin Anggara[1,*], Hannah Ochner[1], Sven Szilagyi[1], Luigi Malavolti[1,*], Stephan Rauschenbach[1,2], Klaus Kern[1,3]

[1]*Max-Planck Institute for Solid-State Research, Heisenbergstrasse 1, Stuttgart, DE-70569, Germany.*
[2]*Chemistry Research Laboratory, Department of Chemistry, University of Oxford, 12 Mansfield Road, Oxford OX1 3TA, United Kingdom.*
[3]*Institut de Physique, École Polytechnique Fédérale de Lausanne, Lausanne, CH-1015, Switzerland.*

Corresponding authors email:

Kelvin Anggara (k.anggara@fkf.mpg.de)
Luigi Malavolti (l.malavolti@fkf.mpg.de)

Supporting Figures

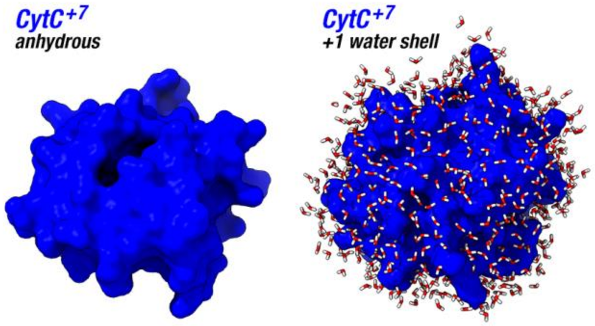

Figure S1 | Computed anhydrous and hydrated protein structures in gas-phase. The anhydrous $CytC^{+7}$ structure (1744 atoms) is computed by relaxing the CytC crystal structure (PDB id: 1hrc) in the gas-phase. The hydrated $CytC^{+7}$ structure (3742 atoms) is computed by relaxing the CytC crystal structure (PDB id: 1hrc) with an addition of ~1 hydration layer of water molecules, prepared by the amorphous ice structure generator, GenIce[60] (https://github.com/vitroid/GenIce).

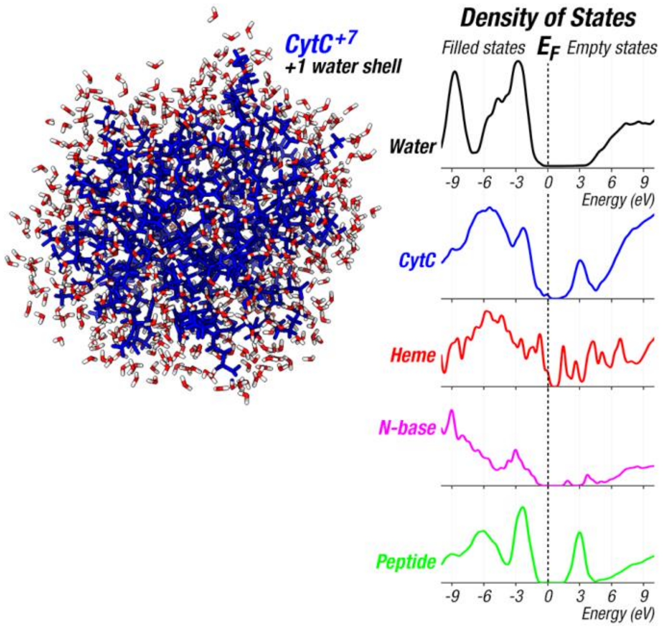

Figure S2 | Electronic structure of hydrated CytC protein. The projected density-of-states (pDOS) of the hydrated CytC is given for the heme (red line), the nitrogen bases (purple line), and the peptide bonds (green line). The pDOS of the whole protein is given in blue line, whilst the pDOS of the water in the hydration shell is given as black line. The pDOS analysis shows that there are CytC electronic states that do not overlap with the water states, specifically those CytC states between 0 to 3 eV above the Fermi level (E_F), which mainly consist of π-states of heme, nitrogen bases, and peptide bonds.

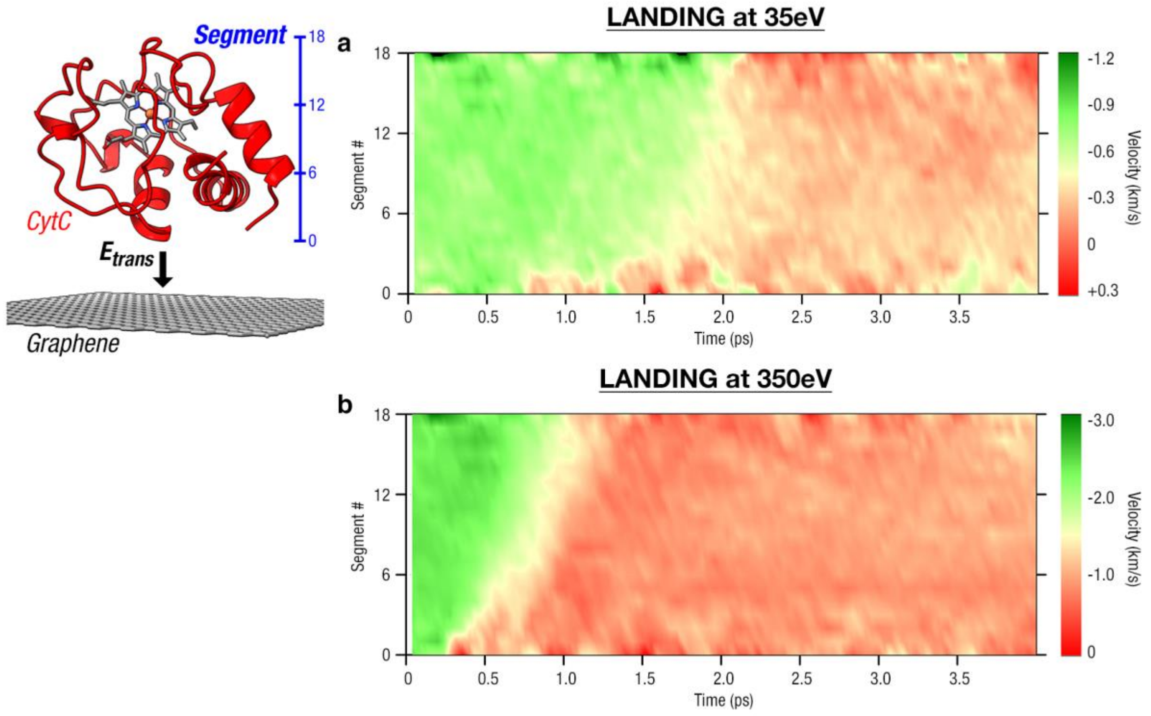

Figure S3 | Quantitative analysis of internal velocities in CytC during its landing on SLG with 35 eV and 350 eV collision energies. The analysis divides the protein along the Z-coordinate to several segments, grouping

the atoms in proteins to respective segments. The plot shows the average velocity of atoms at various segments in the protein during its landing dynamics. The plot shows the speed of soliton propagating internally along the protein as it lands on the SLG.

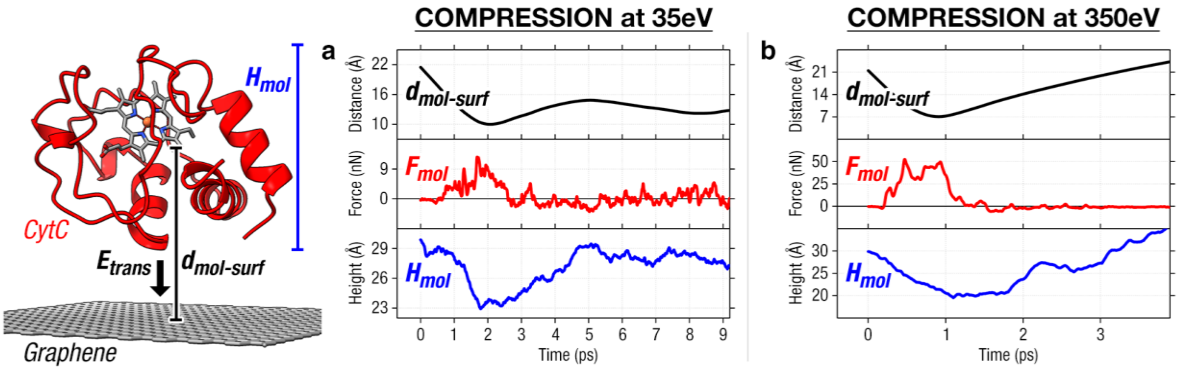

Figure S4 | Quantitative analysis of CytC compression dynamics on SLG with 35 eV and 350 eV collision energies. The analysis shows the time-dependence of molecule-surface distance (dmol-surf), forces acting on molecule (Fmol), and the height of the molecule (Hmol). The molecule-surface distance (dmol-surf) indicates the distance between the CytC center-of-mass and the SLG center-of-mass. The forces acting on molecule (Fmol) is computed by multiplying the CytC center-of-mass acceleration/deceleration with its mass. The height of the molecule (Hmol) is computed by taking the Z-coordinate difference between the protein atom that has the greatest Z-coordinate, and the protein atom that has the lowest Z-coordinate.

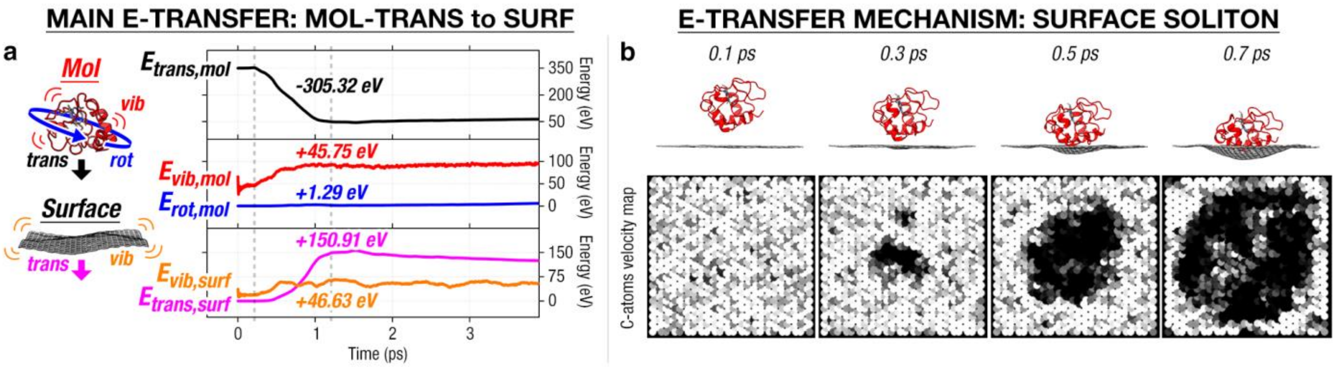

Figure S5 | Quantitative analysis of protein landing dynamics on single-layer graphene at 350 eV collision energy. (a) Protein-SLG collision principally converts the protein translational energy (Etrans,mol) to the SLG vibrational energy (Evib,surf and Etrans,surf). Here the SLG translational energy (Etrans,surf) would approximate the energy received by the fundamental 'trampoline' mode of the SLG. **(b)** Protein-SLG collision creates a soliton in the SLG that serves as a mechanism to promptly transport energy away from the landing site. (black atom = C-atom moving away from protein)

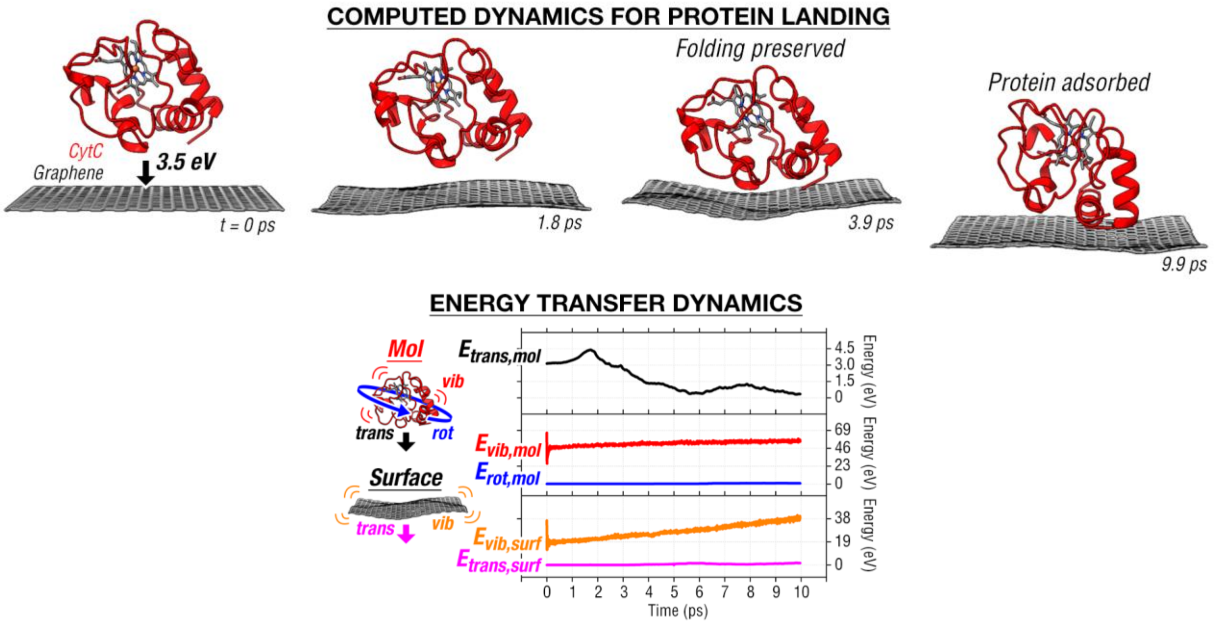

Figure S6 | *Ab initio* molecular dynamics of CytC landing on single-layer graphene at 3.5 eV collision energy. The protein is slightly reoriented prior to its contact with graphene due to attractive forces between the incoming protein and the underlying graphene, evidenced by the increase of translation energy prior to its surface collision (lower panel). The low collision energy preserves the folding state of the protein upon landing, and allows more time for the protein to experience the attractive forces from the surface, which cause the protein to further reorient itself on graphene *en route* towards its most stable adsorption geometry. The graphene has traveled downward by 0.8 nm at the 9.9 ps mark.
