# Supplementary material for: Landing Proteins on Graphene Trampoline Preserves Their Gas-Phase Folding on the Surface
Source: ACS Cent Sci. 2022 Dec 14;9(2):151–8. doi: 10.1021/acscentsci.2c00815 (PMC9951278; doi:10.1021/acscentsci.2c00815)
Supplement: Supplementary file 2 — oc2c00815_si_002.pdf [file oc2c00815_si_002.pdf]

oc-2022-00815t.R1

Name: Peer Review Information for "Landing Proteins on Graphene Trampoline Preserves their Gas-Phase Folding on Surface"

First Round of Reviewer Comments

Reviewer: 1

Comments to the Author

In this manuscript, the authors bring experimental, and mainly modeling / simulation evidence for the role that non-supported 2D materials, in this case, graphene can play as “adapative” support for the deposition of for instance proteins in the gas phase. With low-energy electron holography individual molecules were imaged. In this contribution, the authors highlight the role that soft vibrational modes of the substrate, i.e. trampoline type motion, could play upon impact of the protein with the substrate, in preserving their gas phase folding.

It’s an intriguing concept, that could turn out to become a useful and important protocol for imaging in particular soft and flexible molecules, at the single molecule level, very relevant for systems that cannot be investigated at the ensemble level.

I just have one trivial question. For this particular system, what would have been the impact on the molecular conformation in absence of such soft vibrational modes?

Reviewer: 2

Comments to the Author

The authors have collected a set of high-quality, exciting data, consistent with publication in ACS Central Science. The strongest components of the paper are the actual measured results and the accompanying computations. Revisions to the document are needed to present the work in the proper context. Some conclusions made are overstatements that are inappropriate given the single projectile and single surface type investigated in this work. I recommend revisions throughout the manuscript to highlight what is new about the current work and to remove claims that are inappropriate. These authors and others have landed proteins and measured proteins by LEEH and other single-molecule methods but here the authors are getting dynamics by also including the ab initio computations for a small protein. The paper strongly highlights the role of the graphene surface, but that seems to me a difficult case to make without showing any data for other surfaces.

1) I don’t agree that collision dynamics have been examined mainly on bulk catalytic surfaces (abstract) or that the authors’ previous T-Vsurf results (p. 9) are sufficient as a comparison to the SLG results. There is a large body of work that involves collisions of ions with self-assembled monolayer surfaces

(Cooks, Wysocki, Hanley, Laskin and Futrell, Prell, etc). This includes calculations, e.g., by Bill Hase and others, and experimental results showing high percentage conversions of T-Vsurf WITHOUT the use of a 2D graphene surface. As early as 1987, authors described hydrocarbon self-assembled monolayers as behaving as a soft mattress compared with fluorocarbons behaving more like a hard wall, Phys. Rev. Lett. 58, 1208 (1987). There is also a nice paper on C60 from many years ago that looks a lot like what the authors call soliton behavior (JPC, 1991, 95, 7138).

2) I'm bothered by the overuse of words such as "unique" in the current manuscript when the authors refer to their recent results. The authors attribute their successful soft landing of cytochrome C to "molecule-on-trampoline" dynamics. Many authors have shown successful soft landing of proteins and other molecules on surfaces and they did not use a 2D "trampoline", e.g., Cooks, Benesch and Robinson, recent matrix landing by Coon, recent landing and electron holography reported by Thermo Fisher Scientific at a couple of conferences, others currently doing soft landing.

Specific suggestions:

3) Abstract. Rewrite the abstract to focus on the novel aspects of the current work. If you did not use any other surface, how do you know that you would not have dispersed the collision impact away from the incident protein within a few picoseconds with alternative surfaces? Change complimentary to complementary.

4) p. 2, Intro , rewrite "of how" in first sentence; 3rd sentence, C in Chemistry should be lowercase,; last paragraph "yet unexplored" seems to me to be a gross overstatement

5) throughout: don't use so many acronyms, it is awkward for the reader

6) if you want to call your result "molecule-on-trampoline", at least state that you are not sure whether you might see similar results e.g., on a hydrocarbon self-assembled monolayer.

7) Were results obtained for any additional energies besides 35 and 350 eV?

8) Rachel Loo has written about the role of salt bridges in in vacuo structures – worth citing?

9) The ion mobility CCS calculated by IMPACT differs by 15% from the experimental value and this is stated as "agrees well" with the experimental value. Is there an expectation for how close the IMPACT value should be to the experimental value?

Overall, this is exciting research and will be of interest to the scientific community.

Author's Response to Peer Review Comments:

Please see Response Letter attached below.

RESPONSE TO REVIEWER 1:

(1) *What would have been the impact on the molecular conformation in absence of such soft vibrational modes?*

Response to (1): For protein-graphene collision, we expect the absence of soft 'trampoline' mode of graphene to cause greater changes to the folding state of the landing protein, as a result of increased transfer of incident molecular translational energy (E_{TRANS}) to molecular vibrational energy (E_{VIB}). This trend has been shown computationally by Meroueh *et al* who compare molecules colliding with a *hard* diamond surface and a *soft* self-assembled monolayer (SAM) surface (*J. Am. Chem. Soc.* **124**, 1524 (2002)). We have revised our manuscript to include this discussion (see MS pg 10, ln 206-209).

RESPONSE TO REVIEWER 2:

(1) *I don't agree that collision dynamics have been examined mainly on bulk catalytic surfaces (abstract) or that the authors' previous T-Vsurf results (p. 9) are sufficient as a comparison to the SLG results.*

Response to (1): We agree that our present work of molecular collisions on atomic membrane should be compared to molecular collisions on organic adlayers at bulk surfaces, whose mechanical properties differ significantly from the underlying bulk surfaces. We have revised our manuscript accordingly (see MS abstract; pg 3, ln 61-62; and pg 10, ln 206-209).

(2) *If you did not use any other surface, how do you know that you would not have dispersed the collision impact away from the incident protein within a few picoseconds with alternative surfaces?*

(3) *If you want to call your result "molecule-on-trampoline", at least state that you are not sure whether you might see similar results e.g., on a hydrocarbon self-assembled monolayer.*

Response to (2) to (3): As discussed in our manuscript, molecules colliding on graphene experience a strong coupling between molecular translation mode, graphene out-of-plane 'trampoline' mode, and the transverse soliton mode, which enables the translation of incident molecule to be impulsively converted into the transverse soliton on graphene. Given that 'trampoline' mode is a feature unique to any *freestanding* 2D materials, our work aims to highlight the important consequence of this 'trampoline' mode for molecules colliding on *freestanding* 2D material, using graphene, the most popular 2D material, as an example. Here we use the term 'molecule-on-trampoline' as a shorthand for molecular collision dynamics on *freestanding* 2D material. For molecular collisions on organic adlayers, we agree that the surface modes of the organic adlayers may facilitate similarly effective molecule-to-surface energy transfer. We have revised our manuscript to include these additional discussion (see MS pg 3, ln 61-62; pg 4, ln 74-75; and pg 12, ln 246-249).

(4) *I'm bothered by the overuse of words such as "unique" in the current manuscript when the authors refer to their recent results. The authors attribute their successful soft landing of cytochrome C to "molecule-on-trampoline" dynamics. Many authors have shown successful soft landing of proteins and other molecules on surfaces and they did not use a 2D "trampoline", e.g., Cooks, Benesch and Robinson, recent matrix landing by Coon, recent landing and electron holography reported by Thermo Fisher Scientific at a couple of conferences, others currently doing soft landing.*

Response to (4): We apologize for the misunderstanding caused by the word 'unique'. We use 'unique' in physical context to refer to the specific final states that can only be accessed by the compression dynamics initiated by molecule-surface collisions. These 'unique' final states are potential wells that are inaccessible by conventional thermal chemistry. We have revised the manuscript to eliminate this misunderstanding,

and we completely agree with the reviewer that the use of 2D 'trampoline' is not a requisite for the intact deposition of molecules on surfaces (see MS pg 12, ln 246-249).

(5) *p. 2, Intro , rewrite "of how" in first sentence; 3rd sentence, C in Chemistry should be lowercase,; last paragraph "yet unexplored" seems to me to be a gross overstatement.*

Response to (5): We thank the reviewer for the suggestions. We have revised our manuscript accordingly (see MS pg 2, ln 29-30, 33, and 43), and fixed typos (see MS pg 7, ln 159 and pg 14, ln 289).

(6) *Throughout: don't use so many acronyms, it is awkward for the reader.*

Response to (6): We have replaced 'SLG' with 'graphene' to improve our manuscript readability.

(7) *Were results obtained for any additional energies besides 35 and 350 eV?*

Response to (7): We have computed protein-graphene collision dynamics at a very low energy of 3.5 eV (now added as Fig S6), which leads to soft-landing of the protein on graphene similar to the final state of the 35 eV trajectory (Fig 3A). The low collision energy provides more time for the protein to experience the attractive forces between the protein and the underlying graphene, which leads to significant protein rotations throughout the collision event. These attractive forces are understood to originate from the attraction of the protein charges with their image charges on graphene, as well as the dispersion forces between the protein and the graphene. We have revised the manuscript to include this (see MS pg 10, ln 212-215).

(8) *Rachel Loo has written about the role of salt bridges in in vacuo structures – worth citing?*

Response to (8): We have added the citation to our manuscript (see MS pg 5, line 108).

(9) *The ion mobility CCS calculated by IMPACT differs by 15% from the experimental value and this is stated as "agrees well" with the experimental value. Is there an expectation for how close the IMPACT value should be to the experimental value?*

Response to (9): Turzo *et al* have reported that an average of ~6% error should be expected from IMPACT (*Nat. Commun.* **13**, 4377 (2022)). The difference between theoretical and experimental CCS may be attributed to the finite temperature effects, such as CytC structural dynamics at ~300 K, that have not been taken into account in present DFT calculations. We have revised our manuscript to include this discussion (see MS pg 5, ln 112-114).

<u>RESPONSE TO EDITORIAL REQUESTS:</u>

(1) *SI AU EMAIL: Please include the email address of the corresponding author on the first the Supporting Information, with an asterisk next to their name in the author list.*

(2) *SI PG#S: The supporting information pages must be numbered consecutively, starting with page S1.*

(3) *ABSTRACT: Please make sure the word count does not exceed 200 words.*

(4) *AU EMAIL: Please label as "email."*

(5) *TOC/SYNOPSIS: Please move to the last page of the manuscript, beneath the References.*

Response to (1) to (5): Done.

oc-2022-00815t.R2

Name: Peer Review Information for "Landing Proteins on Graphene Trampoline Preserves their Gas-Phase Folding on Surface"

Second Round of Reviewer Comments

Reviewer: 1

Comments to the Author

The authors have properly revised their manuscript according to the comments.

Reviewer: 2

I remain positive about that manuscript and hope to see it published in ACS Central Science. After reading the manuscript and then the response to the reviews, I have concluded that repeating my original review, with notes that may be useful to both the editor and authors, is the best course of action because those comments still apply. My original review is in red. Comments added on re-review are in blue.

The authors have collected a set of high-quality, exciting data, consistent with publication in ACS Central Science. The strongest components of the paper are the actual measured results and the accompanying computations. Revisions to the document are needed to present the work in the proper context. Some conclusions made are overstatements that are inappropriate given the single projectile and single surface type investigated in this work. I recommend revisions throughout the manuscript to highlight what is new about the current work and to remove inappropriate claims. These authors and others have landed proteins and measured proteins by LEEH and other single-molecule methods but, here, the authors are getting dynamics by also including the ab initio computations for a small protein. The paper strongly highlights the role of the graphene surface, but that seems to me a difficult case to make without showing any data for other surfaces.

After reading the revised manuscript, before (and after) reading the response to reviews, I find that some of the context issues and overstatements persist after revision. This is strong work – why not present it in context and without overstatements inappropriate to the limited dataset presented? I expect to cite this work frequently, but I'd prefer to do that without having to explain the context that the authors did not provide in their writing. It is also simply wrong not to give appropriate literature credit and background.

1) I don't agree that collision dynamics have been examined mainly on bulk catalytic surfaces (abstract) or that the authors' previous T-Vsurf results (p. 9) are sufficient as a comparison to the SLG results. There is a large body of work that involves collisions of ions with self-assembled monolayer surfaces (Cooks, Wysocki, Hanley, Laskin and Futrell, Prell, etc).

Thank you for including some of this work. I still think that Prell's recent SIU paper would be a good addition. The Wysocki group's recent Chem Rev (protein complexes) is more appropriate than the 1996 article (peptides) currently cited.

This includes calculations, e.g., by Bill Hase and others, and experimental results showing high percentage conversions of T-Vsurf WITHOUT the use of a 2D graphene surface. As early as 1987, authors described hydrocarbon self-assembled monolayers as behaving as a soft mattress compared with fluorocarbons behaving more like a hard wall, Phys. Rev. Lett. 58, 1208 (1987). There is also a nice paper on C60 from many years ago that looks a lot like what the authors call soliton behavior (JPC, 1991, 95, 7138).

Thank you for including some Hase work. It is, however, a major omission to leave out the J. Phys. Chem. article that I mentioned in the original review. I am copying below the major figure from that paper, which shows a compression mechanism very similar to the compression mechanism that you mention here (yes, it was C60 and not a small protein but the idea is the same). Because you are championing a "trampoline" mechanism, I think the soft mattress/hard wall citation is also appropriate, especially because groups over the years have compared "hard" fluorocarbon and "soft" hydrocarbon monolayers as collision surfaces for peptides and proteins (Cooks, Wysocki, Hanley).

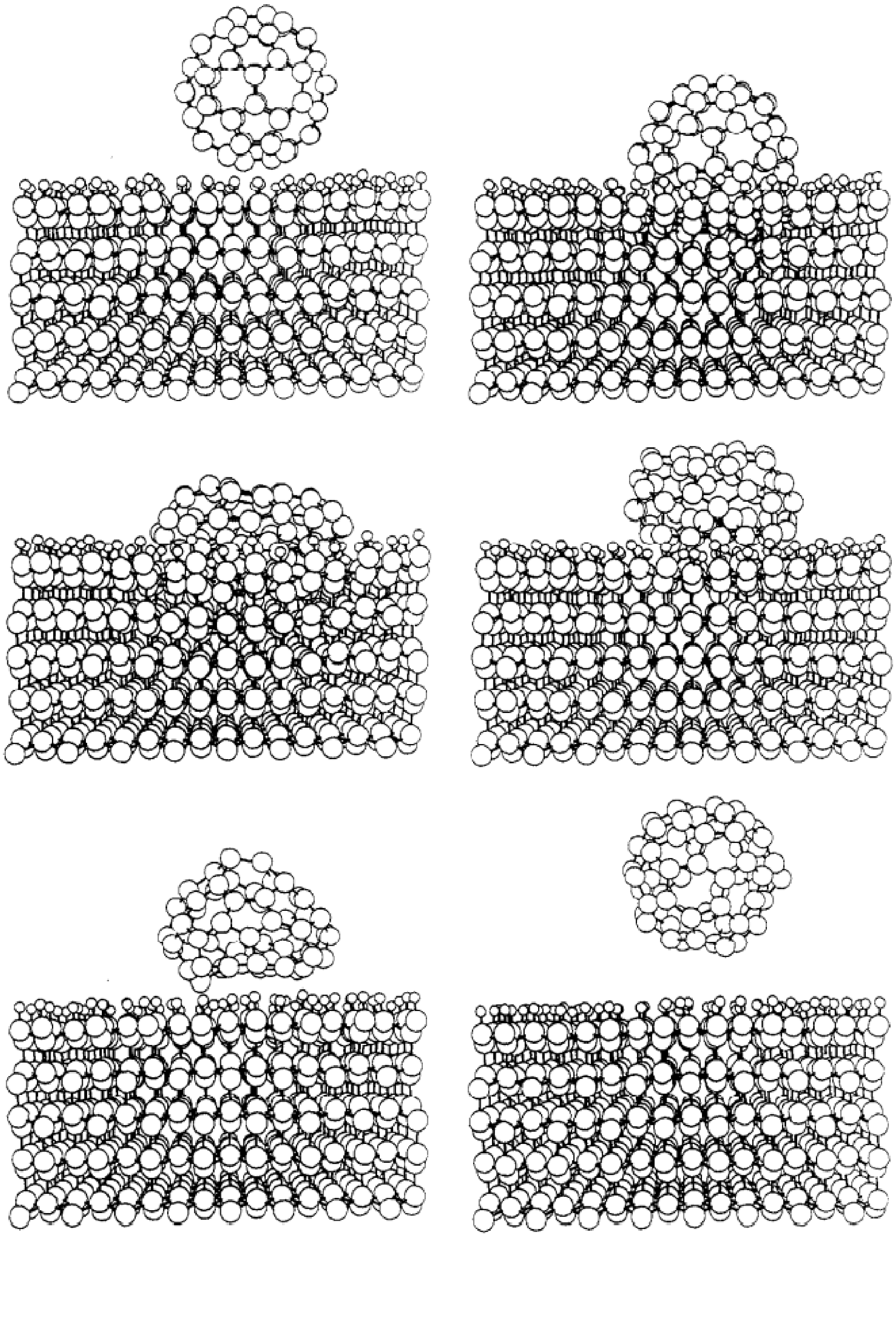

2) I'm bothered by the overuse of words such as "unique" in the current manuscript when the authors refer to their recent results. The authors attribute their successful soft landing of cytochrome C to "molecule-on-trampoline" dynamics. Many authors have shown successful soft landing of proteins and other molecules on surfaces and they did not use a 2D "trampoline", e.g., Cooks, Benesch and Robinson, recent matrix landing by Coon, recent landing and electron holography reported by Thermo Fisher Scientific at a couple of conferences, others currently doing soft landing.

Previous soft-landing results should be presented clearly in this manuscript. Even the work of some of the present authors needs to be mentioned and put in context. This also begs the question of why the authors chose to show results here for only one globular protein. Their previous landing of an antibody, for example, shows the value of choosing a system that is not just globular. While I can understand not presenting ab initio calculations for an antibody, presenting experimental results for a non-globular system on graphene would support the authors' broad claims about the value of "molecule on trampoline".

Specific suggestions:

3) Abstract. Rewrite the abstract to focus on the novel aspects of the current work. If you did not use any other surface, how do you know that you would not have dispersed the collision impact away from the incident protein within a few picoseconds with alternative surfaces? Change complimentary to complementary.

4) p. 2, Intro , rewrite "of how" in first sentence; 3rd sentence, C in Chemistry should be lowercase,; last paragraph "yet unexplored" seems to me to be a gross overstatement.

Thank you.

5) throughout: don't use so many acronyms, it is awkward for the reader

Thank you. The use of graphene vs SLG is an improvement. I am not convinced that ESIBD is needed but it is a minor point.

6) if you want to call your result "molecule-on-trampoline", at least state that you are not sure whether you might see similar results e.g., on a hydrocarbon self-assembled monolayer.

Thank you for including comments about what you are calling organic adlayers.

7) Were results obtained for any additional energies besides 35 and 350 eV?

Thanks for adding material to the SI.

8) Rachel Loo has written about the role of salt bridges in in vacuo structures – worth citing?

Thanks for adding.

9) The ion mobility CCS calculated by IMPACT differs by 15% from the experimental value and this is stated as "agrees well" with the experimental value. Is there an expectation for how close the IMPACT value should be to the experimental value?

I find the temperature argument unsatisfying but at least it offers some speculation about why the numbers disagree.

Overall, this is exciting research and will be of interest to the scientific community.

New comments regarding content in revision:

Fig 2 caption – clarify what is meant by in vacuo structure. Do you mean computed structure? You are assuming that the landed and calculated are the same but that is a stretch based on a similar 2 D shadow for a globular protein.

Figure 3 caption, last line. Clarify distances traveled. 4.7 nm at 3.8 ps – does this mean the greatest distance traveled over the 3.8 ps shown?

Could you mention/name additional, specific 2D freestanding atomic membranes that are alluded to in the text?

"analytical" mentioned in two locations - would be more accurate if replaced with structural biology or macromolecular structure characterization

Fragmentation (or fragmentation of non-covalent complexes) would be a better term/phrase than shattering, if that is what you mean. Shattering is a term that was used in a curious way, mainly by one research group, and later calculations for molecules of any significant size suggested that shattering does not occur. Even the data in the current manuscript is inconsistent with "shattering" – the authors' results show the cyt C traveling away from the surface intact at the higher collision energy.

Paragraph 3 of the intro is one place where better context is needed. There has been progress by multiple groups.

The following sentence (p. 10) is an example of an overstatement. "The T.Vsurf dynamics on freestanding atomic membrane, here termed molecule-on-trampoline dynamics, are expected to be generally operative in the encounter of any molecules with any freestanding 2D materials." You are presenting results for one molecule (a small, globular protein) on one surface. Can you really make this claim based on such limited data? Any molecule of any size? Individual lipid? Any non-covalent complex? Membrane protein in micelle, liposome, nanodisc - will lipids stick, depart, etc? RNA/DNA complexes with proteins?

The last two sentences of the conclusion are another example of this type of overly broad statement. Is this really a "new way"? Any macromolecule?

p. 14, can you be sure that the charge change is a result of e- injection to the protein and not proton transfer to graphene or adsorbates on graphene?

p. 16, top paragraph mentioning surface-collision-induced compression – cite C60 paper; second paragraph energy analysis – instead of citing only your own previous work, cite related work from the literature (Cooks; Hanley, for example) for proper context – those experimental studies are also important later in the paragraph where you cite Hase

Did you try landing denatured cyt C?

Author's Response to Peer Review Comments:

Please see response enclosed.

RESPONSE TO REVIEWER:

(1) *I still think that Prell's recent SIU paper would be a good addition. The Wysocki group's recent Chem Rev (protein complexes) is more appropriate than the 1996 article (peptides) currently cited.*

(2) *Thank you for including some Hase work. It is, however, a major omission to leave out the J. Phys.Chem. article that I mentioned in the original review. I am copying below the major figure from that paper, which shows a compression mechanism very similar to the compression mechanism that you mention here (yes, it was C60 and not a small protein but the idea is the same).*

(3) *Because you are championing a "trampoline" mechanism, I think the soft mattress/hard wall citation is also appropriate, especially because groups over the years have compared "hard" fluorocarbon and "soft" hydrocarbon monolayers as collision surfaces for peptides and proteins (Cooks, Wysocki, Hanley).*

Response to (1 – 3): We thank the reviewer for the suggestions and have added these references to our MS.

(4) *Previous soft-landing results should be presented clearly in this manuscript. Even the work of some of the present authors needs to be mentioned and put in context. This also begs the question of why the authors chose to show results here for only one globular protein. Their previous landing of an antibody, for example, shows the value of choosing a system that is not just globular. While I can understand not presenting ab initio calculations for an antibody, presenting experimental results for a non-globular system on graphene would support the authors' broad claims about the value of "molecule on trampoline".*

(5) *Paragraph 3 of the intro is one place where better context is needed. There has been progress by multiple groups.*

Response to (4 – 5): We agree that different types of proteins have been shown in previous deposition experiments to retain structures, and we agree that presenting these works would underpin our conclusions on the importance of energy transfer from molecular translation to the surface modes. However, as correctly pointed out by the reviewer, the focus of present work on experiment-theory comparison limits the scope to a small globular protein. We have revised our MS to include this discussion (see MS pg 3, ln 52-54).

(6) *Fig 2 caption – clarify what is meant by in vacuo structure. Do you mean computed structure? You are assuming that the landed and calculated are the same but that is a stretch based on a similar 2 D shadow for a globular protein.*

Response to (6): The *in vacuo* structure is the relaxed CytC on graphene computed by our DFT calculations. We have revised the captions to clarify this (see MS pg 6, ln 131-132).

(7) *Figure 3 caption, last line. Clarify distances traveled. 4.7 nm at 3.8 ps – does this mean the greatest distance traveled over the 3.8 ps shown?*

Response to (7): The distance of 4.7 nm is the greatest distance traveled over the 3.8 ps shown. We have revised our MS accordingly (see MS pg 9, ln 193-194).

(8) *Could you mention/name additional, specific 2D freestanding atomic membranes that are alluded to in the text?*

Response to (8): Graphene, due to its mature fabrication technology, remains to be the most popular freestanding atomic membrane used across scientific disciplines, starting from fundamental physics (eg. Cao *et al Nature* **556**, 43 (2018)) to structural biology (eg. Naydenova *et al Proc. Natl. Acad. Sci.* **116**, 11718 (2019)). Emerging freestanding 2D materials includes graphene oxide, hexagonal boron nitride, or

metal chalcogenide monolayer that show diverse physical and chemical properties as outlined by Zhang *et al* (*Nanoscale* **13**, 1443 (2021)). We have revised our MS to include this discussion (see MS pg 4, ln 79-81).

(9) *"analytical" mentioned in two locations - would be more accurate if replaced with structural biology or macromolecular structure characterization.*

(10) *Fragmentation (or fragmentation of non-covalent complexes) would be a better term/phrase than shattering, if that is what you mean. Shattering is a term that was used in a curious way, mainly by one research group, and later calculations for molecules of any significant size suggested that shattering does not occur. Even the data in the current manuscript is inconsistent with "shattering" – the authors' results show the Cyt C traveling away from the surface intact at the higher collision energy.*

Response to (9 – 10): We have revised our MS accordingly (see MS pg 2, ln 34-35; pg 2, ln 42; and pg 10; ln 210).

(11) *The following sentence (p. 10) is an example of an overstatement. "The T.Vsurf dynamics on freestanding atomic membrane, here termed molecule-on-trampoline dynamics, are expected to be generally operative in the encounter of any molecules with any freestanding 2D materials." You are presenting results for one molecule (a small, globular protein) on one surface. Can you really make this claim based on such limited data? Any molecule of any size? Individual lipid? Any non-covalent complex? Membrane protein in micelle, liposome, nanodisc - will lipids stick, depart, etc? RNA/DNA complexes with proteins?*

Response to (11): We agree with the reviewer that it will be interesting to examine the scaling behavior of the dynamics across projectile sizes, starting from small molecules (eg. single lipid) to large macromolecular complexes (eg. protein on micelles). Given that the present work focuses on molecule-on-trampoline dynamics that emerges due to the six to eight orders-of-magnitude difference between vibrational frequencies of the projectile molecule (10^{12} – 10^{14} Hz) and the atomic membrane (10^{6} Hz), we have revised our MS to state that 'the molecule-on-trampoline dynamics are expected to be operative in the encounter of rigid molecules with many freestanding 2D materials' (see abstract; MS pg 4, ln 79; and pg 11, ln 245-246)

(12) *The last two sentences of the conclusion are another example of this type of overly broad statement. Is this really a "new way"? Any macromolecule?*

Response to (12): As per our response to (11), we have revised our MS to state that: 'surface collision on atomic membranes could provide a means to access the ground and excited conformational state of macromolecules via their compressions' (see MS pg 12, ln 258-259).

(13) *p. 14, can you be sure that the charge change is a result of e- injection to the protein and not proton transfer to graphene or adsorbates on graphene?*

Response to (13): Our relaxation calculations show that all protons that are initially attached to the protein in the gas-phase remain attached to the protein when it is fully adsorbed on the graphene. We have revised our MS to include this clarification (see MS pg 8, ln 173-175).

(14) *p. 16, top paragraph mentioning surface-collision-induced compression – cite C60 paper; second paragraph energy analysis – instead of citing only your own previous work, cite related work from the literature (Cooks; Hanley, for example) for proper context – those experimental studies are also important later in the paragraph where you cite Hase.*

Response to (14): We have revised the MS to include these references (see MS pg 10, ln 206-207; and pg 10, ln 215-218).

(15) *Did you try landing denatured Cyt C?*

Response to (15): The landing and imaging of denatured CytC on surfaces have been shown by Deng *et al* (*Nano Lett.* **12**, 2452 (2012)) and Rinke *et al* (*Nano Lett.* **14**, 5609 (2014)).
